# A Systematic Review of the Effectiveness of Concussion Education Programs for Coaches and Parents of Youth Athletes

**DOI:** 10.3390/ijerph17082665

**Published:** 2020-04-13

**Authors:** Robyn Feiss, Molly Lutz, Elaine Reiche, Justin Moody, Melissa Pangelinan

**Affiliations:** 1 School of Kinesiology, Auburn University, Auburn, AL 36849, USA; elaine.reiche@gmail.com (E.R.); mgp0020@auburn.edu (M.P.); 2 Andrews Institute for Orthopaedics & Sports Medicine, Gulf Breeze, FL 32561, USA; mollyroselutz@gmail.com; 3 School of Health Professions, Samford University, Birmingham, AL 35229, USA; jrmoody@samford.edu

**Keywords:** adolescent, children, injury prevention, knowledge transfer, knowledge translation, program evaluation

## Abstract

Coach and parent concussion education programs are essential for the prevention, diagnosis, management, and return to play of youth athletes. This systematic review examined the content and efficacy (changes in knowledge, impact on concussion incidence) of concussion education programs for coaches and parents of youth and high school athletes. Six databases were searched: SPORTDiscus, Academic Search Premiere, CINAHL, PsycINFO, PubMed, and Google Scholar. Studies evaluated the use and/or efficacy of concussion education programs among coaches or parents of youth athletes. A total of 13 articles (out of 1553 articles) met selection criteria. Although different concussion education programs exist, only three have been evaluated in the literature: ACTive Athletic Concussion Training™, USA Football’s Heads Up Football, and the Center for Disease Control and Prevention’s HEADS UP. These programs are well liked among coaches and parents and the suggested practices are easily implemented by coaches. These programs increased concussion knowledge among coaches and parents and promoted behavioral changes among coaches to reduce the concussion risk in high school sports. Few studies have assessed the efficacy of concussion education programs on youth athlete health outcomes. No studies included a longitudinal follow up to determine the degree of knowledge retention following the intervention. While online educational programs are sufficient to improve coach knowledge, in-person training may be a more effective educational tool for reducing the incidence of youth sport concussion. Future studies addressing the efficacy of concussion education programs should include a longitudinal follow up to assess knowledge retention and fidelity.

## 1. Introduction

Sport-related concussions affect an estimated 300,000 children and adolescents in the U.S. annually [1], prompting the rise in research and programs aimed at reducing the incidence rates. Coach education on youth sport concussion (e.g., Center for Disease Control and Prevention’s HEADS UP (CDC HEADS UP), Parachute (formerly ThinkFirst Canada)) provides information regarding general concussion knowledge, recognition of signs and symptoms, and management/treatment. However, the delivery method and specific content differ between programs. The focus of this systematic review is to critically evaluate the content and efficacy of existing programs on coach and parent knowledge to identify the knowledge gaps to guide future program development and research.

Most concussion education programs aim to provide coaches with information on concussion to ensure safe play. Parent-centered education is critically important, as many concussion signs and symptoms may not appear until hours, or even days, following the concussive incident. As such, off the field, the onus often falls on the parents, rather than coaches, to identify signs and symptoms to ensure proper diagnosis, treatment/management, and ensure safe return to play/school [2]. Although programs may include written content directed towards parents and/or encourage parents to access the resources designed for coaches, there are no programs specifically designed for parents.

The purpose of this systematic review was to determine the use and efficacy of current concussion education programs for youth coaches and parents. The goal was to identify effective programs or components of programs, evaluate their influence on coach and parent knowledge of concussion, and determine their impact on youth athlete health outcomes.

## 2. Materials and Methods

### 2.1. Data Sources

Consistent with the PRISMA guidelines [3], six databases were included in the search: SPORTDiscus, Academic Search Premiere, CINAHL, PsycINFO, PubMed, and Google Scholar. The following search terms were used: (inform* OR educat* OR aware* OR know*) AND (parent* OR coach*) AND (concussion OR mTBI OR mild traumatic brain injury) AND sport AND (prevent* OR reduc*).

### 2.2. Inclusion and Exclusion Criteria

**Figure 1:**
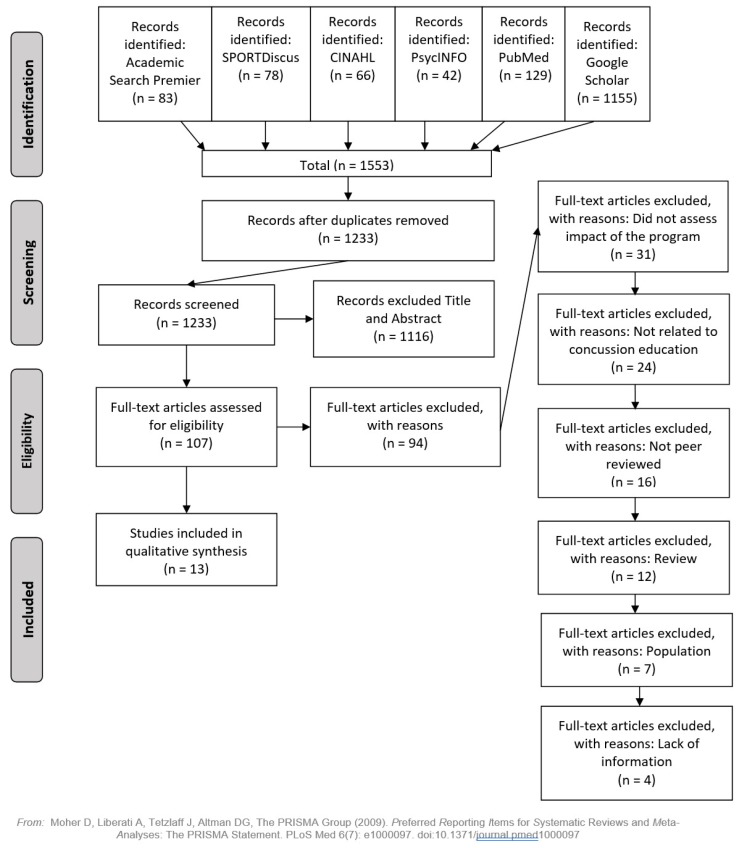
The PRISMA flowchart of the article selection process.

To be included in the review, studies implemented or evaluated a concussion education program with coaches and/or parents and were published in a peer-reviewed journal. No limit was set for the year articles could be published. Figure 1 depicts the PRISMA flowchart, outlining the different stages of the identification and eligibility review. The initial search conducted on December 11, 2019, returned 1553 articles.

### 2.3. Data Extraction

After removing duplicates, 1233 articles were screened by title and abstract. A total of 107 articles were submitted for full-text review—of which, a total of 94 articles were excluded and 13 met inclusion. The title, abstract, and full-text reviews were conducted by RF and ML. The rate of agreement for full-text review was 93.5%, and disagreements were resolved via discussion with JM. The following information was extracted: author names and year of publication, name of the program, number of participants, participant details, delivery method, outcome measures (e.g., change in attitudes/beliefs or concussion knowledge, thoughts/use of program tools, and number of head impacts/concussions), and key findings (see Table 1).

### 2.4. Risk of Bias

**Figure 2:**
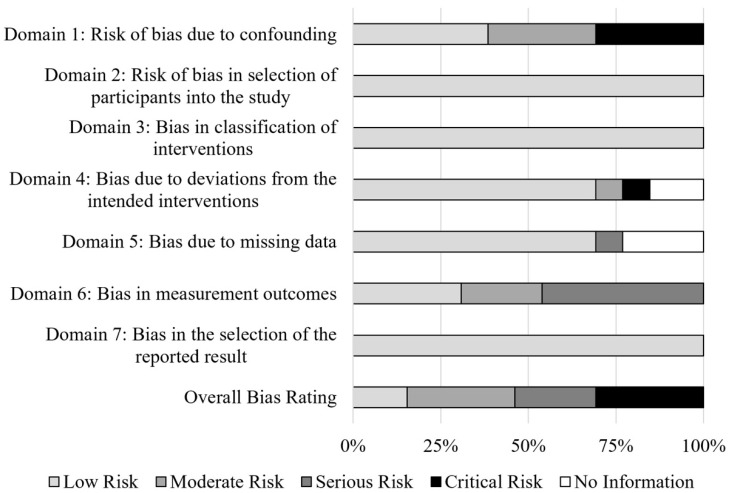
Risk of bias summary graph.

**Figure 3:**
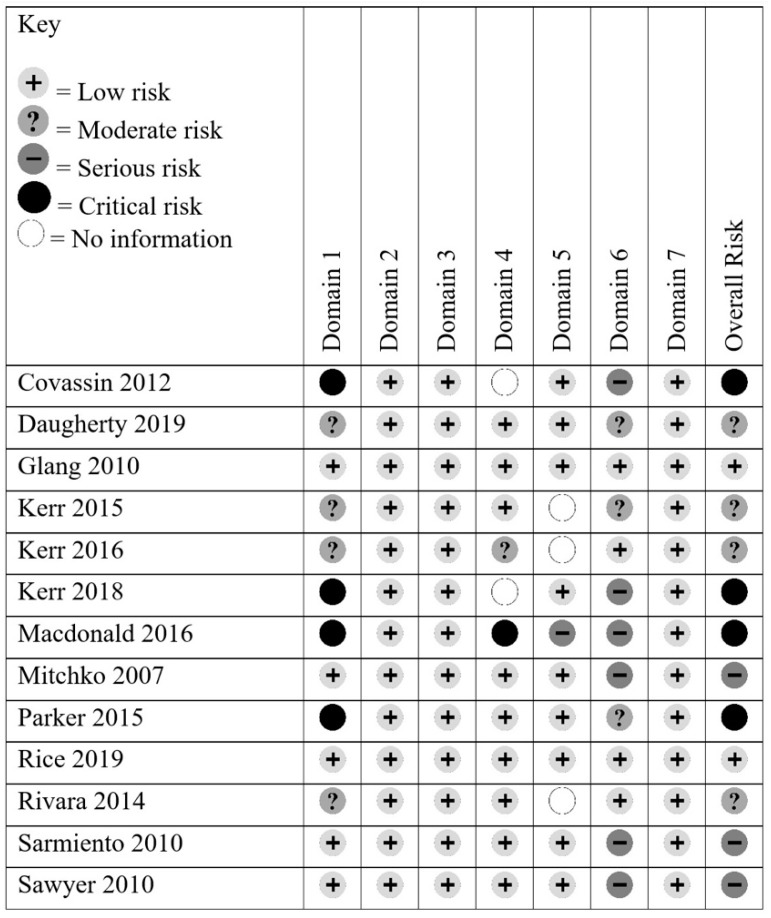
Risk of bias summary chart including judgements regarding risk of bias for all seven.

As only 2 of the 13 studies were RCTs, ROBINS-I [17] was used to assess risk of bias, as this instrument was designed to assess risk of bias in studies with non-randomized controlled designs. Seven different risk bias domains are evaluated: confounding, selection of participants into the study, classification of the interventions, deviations from the intended interventions, missing data, measurement outcomes, and the selection of the reported result. Each study was rated as “low risk”, “moderate risk”, “serious risk”, “critical risk”, or “no information” in each bias domain [17]. An overall risk score was given for each study based on the most serious bias risk in each individual domain. RF and ER assessed risk of bias for each of the included studies. The rate of agreement was 92.3%; disagreement was resolved via discussion. A summary of the bias rankings across all studies is depicted in Figure 2 and individual bias ratings for each study are depicted in Figure 3.

## 3. Results

### 3.1. Data Synthesis

All studies evaluated were published between 2007 and 2019. The results are organized by program: ACTive Athletic Concussion Training™ [18], the CDC HEADS UP [19], and USA Football’s Heads Up Football [20].

### 3.2. Risk of Bias in Included Studies

The 13 studies included in the systematic review were assessed for risk of bias using ROBINS-I [17] (see Figure 2 and Figure 3). Four studies [4,8,10,12] were rated as critical risk for overall bias. This was due to due to the critical risk of confounding, as confounding variables were not often mentioned or controlled for [4,8,10,12]. One study had a critical risk for bias due to deviations from the intended intervention; 40% of the participants did not complete the intervention [10]. However, 9 out of 13 studies were rated as low risk for this category. Nine out of 13 studies had a low risk of bias due to missing outcome data; most studies included data for all participants. Three studies provided no information regarding missing data and were therefore rated as no information for this category [7,9,14]. Six studies were rated as having a serious risk of bias in measurement outcomes; these studies had only one group and the outcome measures could be influenced by the knowledge of the intervention [4,8,10,11,15,16]. Risk rankings for each domain for each article are depicted in Figure 3.

### 3.3. Review of Programs

While there are many concussion education programs available, only three of these programs have been evaluated in the literature: ACTive Athletic Concussion Training™ [18], USA Football’s Heads Up Football (HUF) [20], and the Center for Disease Control’s HEADS UP [19]. These programs deliver content via online training modules to educate coaches and focus on identifying the signs and symptoms of concussion. One program (HUF) offers in-person training in addition to completing online modules. In order to understand the outcomes from each program and differences in coach/parent knowledge resulting from these programs, a brief description of each program (content and format) is provided.

#### 3.3.1. ACTive: Athletic Concussion Training™ for Coaches (ACTive™)

This program is based on recommendations from the National Athletic Trainers’ Association and the International Conference on Concussion in Sport [21,22]. This free program is designed for youth coaches, and consists of a 20 minute interactive video that provides education on concussions, including the signs and symptoms, and how to manage athletes with a suspected concussion [6]. 

#### 3.3.2. USA Football’s Heads Up Football (HUF)

The original HUF program was developed by USA Football and consisted of two key features: a coach certification and a player safety coach (PSC). The coach certification is made up of five online components designed to prevent injury and ensure player safety: concussion recognition and response, equipment fitting, and tackling/blocking techniques related to concussion prevention. The PSC is nominated by the school or league and receives in-person training by a USA Football Master Trainer to ensure that every team correctly implements the HUF health and safety protocols. The PSCs ensure every coach is properly certified and that players, parents, other staff members have the guidelines for adherence of HUF standards throughout the season. The PSC component of the program was discontinued in 2018 and has been replaced by an optional 4 hour in-person clinic for coaches [20].

#### 3.3.3. CDC HEADS UP

The most widely used concussion education programs for coaches are the CDC HEADS UP: Concussion in Youth Sports and HEADS UP: Concussion in High School Sports programs [12,19]. Coaches are provided with an online training course, fact sheet for coaches, a clipboard concussion information sheet, an action plan containing recommended steps following a suspected concussion, a concussion fact sheet for parents, a concussion poster, a signs and symptoms poster, a main message poster, access to an “ask the experts” session on Facebook, and the HEADS UP App on Concussion and Helmet Safety.

#### 3.3.4. Parent Programs

There are no training programs specifically targeting parents of youth athletes designed to increase their knowledge and understanding of concussion. Currently, the CDC HEADS UP program offers a fact sheet for parents, a concussion information sheet for parents and athletes, and access to the HEADS UP App on Concussion and Helmet Safety; these materials provide information on the signs and symptoms of concussion and concussion management. Although parents may complete the HEADS UP program online training course, the program is not specifically designed to address parent-specific knowledge gaps. The information for parents focuses on the definition of a concussion and the signs and symptoms associated with a concussion.

#### 3.3.5. Comparison of Concussion Education Program Content

Table 2 includes additional information regarding the specific content of each program. There are many similarities among the three programs for coaches. All programs use online video components produced with a similar format and interactive quizzes to assess content knowledge and include sections on the definition and mechanism of a concussion, the signs and symptoms of a concussion, proper concussion management, and the return-to-play process following recovery. Additionally, some of the specific materials and content provided by each program are the same. For example, the video for the concussion component of the HUF program is the same video used for the CDC HEADS UP: Concussion in High School Sports program. 

There are some important differences between the programs. For example, the ACTive program discusses concussion rates among different sports and the use of baseline assessments; these topics are not discussed in the other programs. While the ACTive program does discuss the return-to-play process, the HUF and CDC programs provide more details regarding the steps of the gradual return-to-play process recommended for youth athletes.

### 3.4. Evaluation of Eduction Program Materials and Implementation

#### 3.4.1. USA Football’s Heads Up Football

One study used an online survey of coaches’ implementation of the HUF program (*N* = 1312) [8]. Coaches rated themselves as good implementers of the program. However, educating parents (71%) and players (68.5%) about recognizing concussions was less often implemented. Educating coaches (92.3%), removing players suspected of being concussed from play (92.9%), and providing adequate recovery time for concussed players before returning to play (92%) were among the most well-implemented components of the program. Most coaches stated that the PSC was important (75.9%) and reported seeing them on the field on a regular basis during practices (74.1%). The presence of a PSC was associated with a 65% decreased risk of failing to implement the concussion recognition and response component of the program (*OR* = 0.35, *95% CI* = 0.22–0.56) [8]. 

#### 3.4.2. CDC HEADS UP: Concussion in High School Sports

Three studies evaluated the “Heads Up: Concussion in High School Sports” program through assessment of coaches’ appraisals, perceptions, intent to use, as well as use of the toolkit and associated materials [11,15,16]. In one study, prior to receiving the toolkit, one-third of the coaches (*N* = 500) reported that they did not have access to materials regarding concussion prevention and management [11]. The majority of coaches found the toolkit useful and valuable (74%–82%), and reported that they would distribute the information to parents, athletes, and/or school officials [11,15]. Moreover, the signs and symptoms cards were the most commonly viewed material (viewed by 89% of coaches) and 96% reported that they would use the cards. 

There appears to be a disconnect between the coaches’ intent to disseminate and actual dissemination of the information. While 76% of coaches planned to share the information with athletes, coaching staff, and/or parents, only 22% of coaches distributed information from the toolkit to school staff, 7.2% reported distributing the fact sheet for athletes, and 4.4% reported distributing the fact sheet for coaches [16]. Coaches reported being too busy or receiving the materials too late in the year to distribute the information [16]. However, another study reported a much higher distribution percentage, with 68% of coaches reporting that they had educated others about preventing and managing concussions [15]. These discrepancies may be due to when the materials were received by coaches; those evaluated in the Sawyer et al. (2010) [16] study (*N* = 497) received the materials in April 2005 (i.e., close to the end of the school year), while coaches in the Sarmiento et al. (2010) [15] study (*N* = 333) received the materials between September 2005 and July 2006 (i.e., during the school year). 

Coaches report changing their behaviors based on program information. Eighty-one percent of coaches whose schools had a previously existing plan to prevent or manage concussions felt that the toolkit would improve the existing plan. Sarmiento et al. (2010) [15] and Sawyer et al. (2010) [16] reported that the toolkit increased prevention and improved management of concussion in high school athletes. Specifically, 34% of coaches reported learning something new from the toolkit. Moreover, those considered “high implementers” (i.e., used four or more of the materials) were more likely to have learned something new than “low implementers” (i.e., used three or less of the materials) [15]. High implementers were also more likely to have reported making changes in the ways they prevent and manage concussions [15].

#### 3.4.3. CDC HEADS UP: Concussion in Youth Sports

One study examined the use of the CDC HEADS UP: Concussion in Youth Sports toolkit [4] and reported that 69.6% of youth coaches surveyed (*N* = 340) did not have access to other concussion materials before receiving the CDC’s toolkit. Coaches reported the fact sheet for coaches (65.7%) and the magnet (63.8%) were the most useful materials in the toolkit. Once provided with the information, 77% of coaches reported being able to more easily identify suspected concussions. Moreover, 71.7% reported educating others regarding concussion prevention or management; however, no details were provided regarding what information was disseminated.

#### 3.4.4. Parent Interventions

Only one study investigated parent feedback regarding concussion education materials [10]. Parents were provided with three educational tools: the CDC Heads Up to School: Know Your Concussion ABC’s handout, CDC HEADS UP Concussion: Online Concussion Training, and an educational YouTube video. Parents reported that the online training provided the most important information, was the most well liked and most attention grabbing, had the greatest impact, and was the least confusing of the three tools. Parents felt that the handout was written for them and was considered the most likely tool to motivate them to seek out further information regarding concussions [10].

### 3.5. Evaluation of Eduction Program Effectiveness

#### 3.5.1. ACTive: Athletic Concussion Training™ for Coaches (ACTive™)

One study evaluated the effectiveness of the ACTive™ [18] online program among coaches (*N* = 75) [6]. The program improved coaches’ general knowledge of concussion by 41%, recognition of signs and symptoms by 37.5%, and reduced common misperceptions by 13.7%. Additionally, coaches reported greater confidence in responding appropriately and an increased intention to act based on possible concussion scenarios after the concussion training [6]. However, this study did not evaluate whether coach education influenced player safety and concussion incidence. No studies have evaluated the ACTive program with parents of youth athletes. 

#### 3.5.2. CDC HEADS UP Concussion in Programs for Coaches

Two studies investigated the impact of concussion education programs on coach knowledge [5,12]. Parker et al. (2015) [12] examined the change in coach knowledge regarding causes, risks, and management of concussions after participating in the CDC’s online course “Concussion in Sports: What You Need to Know”. Over 98% of coaches were already aware that a concussion is a traumatic brain injury that can interfere with normal brain function (*N* = 132,312/133,764) and that returning to play too soon could increase risk of a second concussion (*N* = 132,494/133,764) before completing the course. The largest change in coach knowledge (from 24.2% to 61.6% of participants) was how to decrease the risks of death or long-term problems from a concussion. Daugherty et al. (2019) [5] examined changes in coaches concussion knowledge, attitudes, and behavioral intentions after participating in the CDC HEADS UP: Concussion in Youth Sports online training program (*N* = 179,469). During pre-test, over 88% of coaches correctly answered the questions of least difficulty (i.e., True/False: A concussion is a brain injury) and low difficulty (i.e., True/False: Athletes should have more than one concussion symptom before they are removed from play). Therefore, limited improvement was observed for these questions. However, larger improvements were seen for questions of moderate (i.e., Most athletes with a concussion feel better (in what time frame)?) and high difficulty (i.e., What percentage of athletes do researchers think try to hide their concussion symptoms from their coach?). Increases in moderate effect sizes were observed for both questions of moderate difficulty (r = −0.48) and high difficulty (r = −0.42), while small effect sizes were observed for questions of the least difficulty (r = −0.12) and low difficulty (r = −0.14). After the training, coaches reported being more confident in their ability to recognize concussion symptoms in youth athletes (r = −0.59) and being more confident in their ability to help with the return-to-play process (r = −0.56). Importantly, coaches also expressed greater intention to speak with their athletes about concussion and encourage them to report concussion symptoms (r = −0.52) [5].

#### 3.5.3. CDC Concussion Education Programs—Parent Outcomes

Two studies investigated the impact of the CDC concussion education programs on parental knowledge [10,13]. Macdonald and Hauber (2016) [10] examined parental knowledge of concussion, information-seeking behavior, and the effectiveness of three education tools for parents (*N* = 29). All three tools improved parent perception, awareness, and general knowledge of concussions. However, the online CDC training had the greatest impact, producing the largest changes in perception, awareness, and parental knowledge of concussion [10]. Rice and Curtis (2019) [13] examined changes in parental knowledge following viewing of the CDC Concussion Awareness Video or the CDC Concussion Fact Sheet for Parents (*N* = 140). The video and the fact sheet produced similar changes in knowledge. The largest improvements were regarding awareness of second impact syndrome (27.1% increase), awareness of the CDC’s “Heads Up: Concussion in Youth Sports” program (27.1% increase), perception that concussions are a critical issue (16.5% increase), and ability to determine when their child was ready to return to sport after a concussion (13.6% increase) [13]. However, parents disagreed that most concussions are preventable (42.9% disagreed or were unsure) and believed that CT and MRI scans can diagnose concussions (67.2%). Overall, parents demonstrated good recognition of signs and symptoms, but had difficulty identifying symptoms related to mood and sleep changes, compared to cognitive or physical symptoms [13].

### 3.6. Educational Programs and Concussion Epidemiology

#### 3.6.1. USA Football’s Heads Up Football

Two studies used randomized designs to examine the influence of the HUF program coach education on the incidence of concussion in youth athletes [7,9]. Kerr et al. (2015) [9] (*N* = 70) found that players whose coaches who completed the HUF education program experienced 24% fewer head-impacts above 10Gs, compared to those whose coaches received no educational training. All six concussions reported during the season occurred in athletes whose coaches had not received training [9]. A follow-up study of 6 teams (*N* = 390 players), found that players whose coaches completed both the online and PSC programs experienced significantly fewer concussions throughout the season than those whose coaches only participated in the online training (Injury Rate Ratio = 0.12) [7]. 

#### 3.6.2. Non-Specified Programs for Coaches

Rivara et al. (2014) [14] assessed the relationship between concussion education modality (i.e., written, video, PowerPoint, quiz, and in-person training) and coach awareness of concussion incidence for football and female soccer coaches. The type of required education for coaches influenced awareness of a player’s concussion—coaches who used the video or quiz were significantly less likely to be aware of their athletes’ concussions than coaches that completed written, PowerPoint, or in-person trainings [14].

## 4. Discussion

Though many concussion education programs exist, only three programs have been evaluated in the literature: ACTive Athletic Concussion Training™ [18], USA Football’s Heads Up Football [20], and the Center for Disease Control’s HEADS UP [19]. These programs are well liked among coaches and parents and are generally well implemented among coaches. These programs increased concussion knowledge among coaches and parents and promoted behavioral changes among coaches that could reduce the concussion prevalence in high school sports. Several studies investigated interest in and the usefulness of concussion education tools, as well as differences in retrospective knowledge of concussion. However, very few studies have investigated real-time differences in safe coaching practices after having participated in an educational training. Additional research is needed to determine the most effective form of concussion education for both coaches and parents, as well as determine changes in concussion incidence and player safety following concussion education interventions.

### 4.1. Program Content

All programs included content on the definition and mechanism of a concussion, the signs and symptoms of a concussion, proper concussion management, and the return-to-play process. However, many of the programs include little or no content regarding emotional symptoms, sleep disturbances, and specific details for youth athletes. This may explain knowledge gaps in these topic areas discussed by Feiss et al. (2020) [2] and suggests that all programs need to include additional information to reduce these specific knowledge gaps. Interestingly, the video for the concussion component of the HUF program is the same video used for the CDC HEADS UP: Concussion in High School Sports program, indicating that there is overlap between the programs. The major differences between the programs involve the emphasis of particular topics. For example, the ACTive™ program includes discussions of concussion rates among different sports and the use of baseline assessments—neither of which are included in the other two programs. The HUF and CDC programs discuss the gradual return-to-play process in much more detail than the ACTive™ program. Lastly, the CDC HEADS UP for High School Sports and the blocking/tackling component of the HUF program provide sport-specific recommendations to reduce concussion incidence; the other two programs do not contain this information. It is unclear whether these program differences influence coach knowledge or concussion incidence among youth athletes.

As previously discussed, there are no concussion education programs specifically designed for parents, although parents can view the video programming designed for coaches. The CDC HEADS UP materials for parents include written materials that are similar in content to the other CDC materials but with less detail and focuses on the signs and symptoms of concussion and concussion management. However, it does not include information on sleep symptoms, greater recovery time for youth athletes, or the higher risks for youth athletes. More information is needed regarding these and other topics that are particularly relevant to and observed by parents.

### 4.2. Use and Implementation of Concussion Education Programs by Coaches

The CDC HEADS UP education programs were well received by coaches at both the youth and high school levels [4,15,16]. The signs and symptoms cards and information booklets were reported to be the most useful materials provided by the CDC HEADS UP program to the high school coaches [15,16], while the fact sheet for coaches was the most useful for youth coaches [4]. These research findings are important for the development and improvement of concussion education materials to address coach knowledge gaps.

One study assessed the implementation of the HUF program [8]. Coaches implemented the program well and the presence of a PSC decreased risk of failing to implement proper concussion recognition and response protocols. However, it is important to note that the coaches rated themselves on their own implementation of the HUF program protocols, which may introduce bias.

### 4.3. Influence of Education Programs on Coach Knowledge

Studies suggests that exposure to both the ACTive™ and CDC HEADS UP programs increased coaches’ general knowledge regarding concussion as well as their knowledge regarding signs and symptoms, management, and the return-to-play process [5,6,12]. Furthermore, this greater knowledge has been associated with viewing concussions more seriously, as well as affecting coach attitudes and beliefs regarding concussions [4,5]. However, there is evidence of a ceiling effect regarding the impact of educational programs [5]. Additionally, no standard questionnaire exists to assess coach knowledge regarding the various concussion topics, which may lead to discrepancies across studies. Future research is needed to develop a standard questionnaire to assess coach knowledge that can be used in various settings as well as determine what aspects of concussion knowledge and education are most important for coaches.

### 4.4. Education Programs for Parents

Although concussion education delivered in various formats improves parent knowledge, perception, and awareness of concussion [10,13], parents continue to express difficulty identifying mood- and sleep-related symptoms compared to cognitive or physical symptoms [13]. Although parents preferred online concussion training to a written handout or YouTube video [10], these findings suggest that the modality of parent education materials may not influence parental knowledge and that educational messaging can be distributed to parents in various forms. However, educational materials need to include mood- and sleep-related symptoms to address parent knowledge gaps. Given the limited number of studies examining the efficacy of educational program on parent knowledge of concussion and a dearth of programs specifically designed for parents, additional research is necessary to investigate changes in parental knowledge following education interventions.

### 4.5. Coach Concussion Education and Youth Athlete Health Outcomes

Kerr et al. (2015, 2016) [7,9] suggest that while the HUF education program may reduce concussion incidence compared to no education, the online component alone was less effective than when combined with an in-person training session for a PSC. Additionally, Rivara et al. (2014) [14] suggests that coaches who have used videos or a quiz to complete their concussion education requirements are less likely to be aware of their athletes’ concussions than those who used a written, PowerPoint, or in-person training. These results are supported by Kerr et al. (2018) [8], who reported that the presence of the PSC increased the likelihood of implementing the concussion recognition and response component of the online training. Together, these studies suggest that while online training may be sufficient to increase coach knowledge of concussion, in-person training may be more effective in reducing concussion incidence among youth athletes. More research is needed to assess changes in practice implementation and the incidence of concussion with currently available concussion education programs. Direct comparison of education programs should be investigated to determine which is the most effective in reducing concussion incidence and improving youth and high school athlete health.

### 4.6. Limitations and Future Directions

This systematic review was limited by the number of studies that met inclusion, the study design implemented, and the methods these studies used to evaluate program efficacy. Five studies assessed a change in knowledge due to educational program interventions using a pre- and post-test assessment [5,6,10,12,13], while others only assessed knowledge levels after providing an educational program [4,8,11,15,16]. No studies included a longitudinal follow up to assess retention of information. Moreover, a standardized assessment is needed measure the same content areas across studies/programs and across time. Efforts are needed to determine what components of education are the most relevant to parents and coaches, as these may differ between the two groups. In addition, studies should determine which delivery methods are the most effective for each group. While online education may be sufficient for parents [10,13], in-person training may be necessary for coaches [7,8]. Additional in-person training for coaches may be necessary to elicit better outcomes for athletes [7,9].

Three studies addressed whether coach education translates to better health outcomes for athletes [7,9,14] and provide preliminary evidence that concussion education for coaches, particularly in-person trainings, can lead to reduced concussion risk. However, more rigorous study designs are needed to determine causal effects. No studies have evaluated the efficacy of the CDC HEADS UP program on reducing concussion incidence, although it is the most commonly program. Longitudinal studies examining all programs are needed to determine which are the most effective in reducing concussion incidence and improving youth and high school athlete health.

Eleven of the 13 studies included in this review exhibited some risk of bias; much of the bias was due to a lack of information or lack of clarity about the information provided. Many studies did not discuss possible confounding variables (e.g., sport coached, environment (urban vs. rural), SES, etc.) and/or used measures that are easily biased. Descriptive statistics were missing from many of the studies, precluding the ability to conduct a meta-analysis to compute effect sizes across studies. Future studies should include descriptive statistics and key study details to enable better assessment of the research quality and allow for future meta-analyses. The risk of bias assessment used was designed to assess risk of bias in non-randomized controlled study designs. However, some studies included in this review implemented one-group designs [4,8,11,15,16]. Due to this limitation, and the small number of studies that met inclusion, studies with critical bias ratings were evaluated presently. Lastly, 11 of the 13 studies were observational or used non-randomized experimental designs. RCTs should be used to reduce risk of bias and strengthen the literature surrounding concussion education interventions.

Although this systematic review addressed important gaps in the literature, there are limitations that should be addressed in future studies. The main limitation is the lack of a comparison of effectiveness across program, which was not possible because of inconsistencies in the reporting of knowledge change (e.g., different categories of knowledge, mean change vs. pre- and post-exposure scores). Future studies addressing knowledge change due to educational interventions should seek to standardize categories of concussion knowledge and provide pre- and post-test knowledge scores to allow for future meta-analyses. Lastly, it is important to note that the risk of bias assessment used presently, ROBINS-I [7], is designed to assess risk of bias in studies with interventions that use non-randomized controlled study designs. However, not all studies evaluated used non-randomized designs, which may have impacted their risk of bias rating. Future studies should employ rigorous study designs (e.g., randomized designs) to reduce bias. 

## 5. Conclusions

Although many concussion education programs exist, three concussion education programs for coaches and parents have been evaluated in the literature. Overall, these programs were well received by both parents and coaches and increased concussion knowledge. Some studies have shown that these programs may also reduce negative health outcomes for youth athletes (i.e., number of head impacts and concussions).

While online educational programs are sufficient to improve coach knowledge, in-person training may be a more effective educational tool for reducing the incidence of youth sport concussion. Although these educational programs are useful for both coaches and parents, the content of these programs should continue to be adjusted based on parent and coach feedback and knowledge gaps reported by high-quality research studies. Lastly, future studies addressing the efficacy of concussion education programs should include a longitudinal follow up to determine retention of the information. This information could be particularly useful in developing efficient “refresher” courses for veteran coaches.

## Figures and Tables

**Table 1:** Study Characteristics and Key Findings Regarding the Effectiveness of Concussion Education Programs (*N* = 13).

References	Educational Program	Number of Participants, Participant Details	Format, Number of Sessions	Outcome Measures	Key Findings
Covassin et al., 2012 [4]	CDC HEADS UP: Concussion in Youth Sport	340, youth sport coaches	Online survey, 6 months after receiving CDC materials	Attitudes/beliefs about concussion; post-concussion actions taken by coaches; opinion/use of CDC materials	After viewing educational materials, the majority of coaches reported viewing concussions as a serious or very serious issue and took the proper actions after an athlete was suspected of a concussion; fact sheet for coaches was the most useful material
Daugherty et al., 2019 [5]	CDC HEADS UP: Concussion in Youth Sport	179,469, youth and high school sport coaches (154,807 youth, 13,043 high school, 11,619 both)	Online surveys, pre- and post-exposure to CDC HEADS UP online training	General concussion knowledge; concussion management; attitudes/beliefs about concussion; intention and self-efficacy in concussion management and prevention	Coaches showed improved knowledge scores after reviewing the material in the online program, particularly for the moderate- and high-difficulty knowledge questions; coaches felt more confident in their ability to recognize concussion symptoms in youth athletes and help an athlete with the return-to-play process; coaches reported being more likely to talk with their athletes about concussions
Glang et al., 2010 [6]	ACTive: Athletic Concussion Training using Interactive Video Education	75, youth sport coaches (40 treatment, 35 control)	RCT, online survey, pre- and post-exposure to ACTive program materials	General concussion knowledge; concussion management; attitudes/beliefs about concussion; intention and self-efficacy in concussion management and prevention	Coaches who were exposed to the ACTive program materials increased their general knowledge, signs and symptoms knowledge, knowledge of misperceptions, and reported greater self-efficacy and behavioral intention to respond correctly to possible concussions
Kerr et al., 2016 [7]	Heads Up Football (HUF)	6, high school football teams	Compared teams with and without the HUF programs player safety coach (PSC) component	Number of concussions	Players on teams whose staff received only the online education experienced more concussions than those whose staff received the full program including in-person training
Kerr et al., 2018 [8]	Heads Up Football (HUF)	1316, football coaches	Online survey, interactions with the player safety coach, implementation of the HUF educational materials	Player safety coach on-site presence during practices; coaches rated their implementation of HUF educational program components	Player safety coach was thought to be important, but rarely seen at practice; coaches implemented most aspects of the program but some did not educate players regarding recognizing concussions
Kerr et al., 2015 [9]	Heads Up Football (HUF)	15, youth football teams, players ages 8–15 (mean age = 11.7)	Compared teams with and without exposure to HUF program and a player safety coach (PSC)	Number of head impacts; number of concussions	Players on teams whose staff had been exposed to HUF training and had a PSC experienced significantly less head impacts during practice than those whose coaches had not completed the training
Macdonald and Hauber, 2016 [10]	CDC HEADS UP Concussion Online Training; CDC HEADS UP to School: Know Your Concussion ABCs; Know the Impact: Concussion Awareness	29, parents of high school football players	Paper survey, post-exposure to all three educational tools	Impact of each educational tool on concussion knowledge, perception, and awareness	CDC HEADS UP Concussion Online Training had the greatest influence on concussion knowledge, perception, and awareness, but was also ranked as the most confusing; CDC HEADS UP to School: Know Your Concussion ABCs was ranked as the tool most seemingly targeted for parents; most parents had no desire to seek out more information regarding concussion after the study
Mitchko et al., 2007 [11]	CDC HEADS UP: Concussion in High School Sports	500, high school coaches	Online survey	Initial evaluation of the CDC HEADS UP Concussion in High School Sports toolkit	The majority of coaches found the toolkit useful; 1/3 of coaches did not have access to concussion education materials prior to receiving the toolkit
Parker et al., 2015 [12]	CDC HEADS UP Concussion in Sports: What You Need to Know online course	133,764, coaches who completed the CDC online course	Online survey, pre- and post-exposure to online educational course	General concussion knowledge; concussion management, diagnosis, and return to play	At least 86% of coaches correctly answered questions related to general knowledge, management, and return to play on the pre-test, increasing to 97.5% on the post-test; greatest improvement was seen in diagnosis as scores increased over 37% from pre- to post-test
Rice and Curtis, 2019 [13]	CDC HEADS UP Concussion Awareness Video and Concussion Fact Sheet for Parents	140 parents of youth club athletes	RCT, online survey pre- and post-exposure to one of the intervention materials	General concussion knowledge; signs and symptoms of concussion, and concussion management	Parent knowledge improved similarly with both interventions; parents successfully identified most signs and symptoms, but also often identified “red herring” signs as true signs of concussion and struggled to identify mood- and sleep-related symptoms; pre-test score and receiving concussion information prior to the intervention were the strongest predictors of post-test knowledge
Rivara et al., 2014 [14]	Multiple modalities (written, video, PowerPoint, 6-item quiz, and in-person training)	40, high school football and women’s soccer coaches; 778, parent–player dyads on coaches’ teams	Coach survey, parent/player phone call to report number of practices/games players participated in, if they had suffered a hit associated with concussive symptoms, their coach’s awareness of the concussion	Coach awareness of concussion; education modality coach was exposed to; number athletes playing with symptoms	Coaches using the video or quiz were 50% or 40% less likely to be aware of concussion; no difference in awareness between number of modalities coach was exposed to; 69% of athletes reported playing with concussive symptoms
Sarmiento et al., 2010 [15]	CDC HEADS UP: Concussion in High School Sports	333, high school coaches	Paper survey	Use of toolkit; knowledge, attitudes, and behaviors towards concussion prevention	The booklet was most used tool; coaches, especially those who implemented multiple aspects of the toolkit, reported learning something new about concussions, regarding concussions more seriously, and changing the waythey prevent or manage concussions
Sawyer et al., 2010 [16]	CDC HEADS UP: Concussion in High School Sports	497, high school coaches	Telephone survey, 2–7 weeks after receiving educational toolkit	Use of/plans to use the toolkit materials; overall opinions of the toolkit	Overall, coaches had either used or planned to use the majority of the materials in the toolkit and found it to be visually appealing and easy to use

**Table 2:** Comparison of Concussion Education Program Content.

Content	ACTive		USA Football’s Heads Up Football		CDC HEADS UP to Youth Sports		CDC HEADS UP to High School Sports		CDC HEADS UP to Parents
	Video	Written Materials	Concussion Video	Blocking/Tackling Video	Video	Written Materials	Video	Written Materials	Written Materials
**General Information**									
Injury does not always occur via direct hit to head	✓	—	—	—	✓	—	—	—	—
Injury mechanism	✓	—	✓	—	✓	✓	✓	✓	✓
How a concussion is diagnosed	—	—	—	—	✓	—	—	—	—
**Signs and Symptoms**									
Cognitive	✓	✓	✓	—	✓	✓	✓	✓	✓
Physical	✓	✓	✓	—	✓	✓	✓	✓	✓
Emotional	✓	✓	✓	—	✓	✓	✓	✓	✓
Sleep	✓	✓	✓	—	—	—	✓	—	—
Loss of consciousness is not necessary for diagnosis	✓	—	✓	—	✓	—	✓	—	—
**Concussion Management**									
Risks of second concussion	✓	—	✓	—	✓	✓	✓	✓	✓
Longer recovery time for youth athletes	✓	—	✓	—	—	—	✓	—	—
Higher risk for youth athletes	✓	—	✓	—	✓	—	✓	—	—
Signs emergency care is required	✓	✓	✓	—	✓	✓	✓	✓	✓
**Return to Play**									
An athlete suspected of a concussion must be immediately removed from play	✓	✓	✓	—	✓	✓	✓	✓	✓
A concussed athlete must be cleared by a physician to begin the return-to-play process	✓	✓	✓	—	✓	✓	✓	✓	✓
Gradual return-to-play process	✓	✓	✓	—	✓	✓	✓	✓	✓
**Sport-Specific Recommendations**	—	—	—	✓	—	—	—	✓	—

Note: A checkmark (✓) indicates that this information was included in the content and an em dash (—) indicates this information was not included in the content.
